# miR-340 predicts glioblastoma survival and modulates key cancer hallmarks through down-regulation of *NRAS*

**DOI:** 10.18632/oncotarget.6968

**Published:** 2016-01-21

**Authors:** Danilo Fiore, Elvira Donnarumma, Giuseppina Roscigno, Margherita Iaboni, Valentina Russo, Alessandra Affinito, Assunta Adamo, Fabio De Martino, Cristina Quintavalle, Giulia Romano, Adelaide Greco, Ylermi Soini, Arturo Brunetti, Carlo M. Croce, Gerolama Condorelli

**Affiliations:** ^1^ Department of Molecular Medicine and Medical Biotechnology, “Federico II” University of Naples, Naples, Italy; ^2^ IRCCS-SDN, Naples, Italy; ^3^ IEOS, CNR, Naples, Italy; ^4^ Institute of Pathology, Molecular Pathology Division, University of Basel, Basel, Switzerland; ^5^ Department of Molecular Virology, Immunology and Medical Genetics, Human Cancer Genetics Program, Comprehensive Cancer Center, The Ohio State University, Columbus, OH, USA; ^6^ Department of Advanced Biomedical Science, University of Naples Federico II, Naples, Italy; ^7^ Ceinge, Biotecnologie Avanzate, Scarl, Naples, Italy; ^8^ Cancer Center of Eastern Finland, University of Eastern Finland, Kuopio, Finland

**Keywords:** microRNAs, glioblastoma, survival, NRAS, signal-transduction

## Abstract

Glioblastoma is the most common primary brain tumor in adults; with a survival rate of 12 months from diagnosis. However, a small subgroup of patients, termed long-term survivors (LTS), has a survival rate longer then 12–14 months. There is thus increasing interest in the identification of molecular signatures predicting glioblastoma prognosis and in how to improve the therapeutic approach. Here, we report miR-340 as prognostic tumor-suppressor microRNA for glioblastoma. We analyzed microRNA expression in > 500 glioblastoma patients and found that although miR-340 is strongly down-regulated in glioblastoma overall, it is up-regulated in LTS patients compared to short-term survivors (STS). Indeed, miR-340 expression predicted better prognosis in glioblastoma patients. Coherently, overexpression of miR-340 in glioblastoma cells was found to produce a tumor-suppressive activity. We identified *NRAS* mRNA as a critical, direct target of miR-340: in fact, miR-340 negatively influenced multiple aspects of glioblastoma tumorigenesis by down-regulating *NRAS* and downstream AKT and ERK pathways. Thus, we demonstrate that expression of miR-340 in glioblastoma is responsible for a strong tumor-suppressive effect in LTS patients by down-regulating NRAS. miR-340 may thus represent a novel marker for glioblastoma diagnosis and prognosis, and may be developed into a tool to improve treatment of glioblastoma.

## INTRODUCTION

Malignant glioma (glioblastoma or GBM) is the most common and aggressive primary brain tumor [1, 2]. Despite continuous improvement in treatment approaches, with a combination of surgery, radiotherapy and chemotherapy, average survival of GBM patients has improved only slightly [3]. In fact, GBM patients tend to have an extremely poor prognosis, with a median survival rate from diagnosis ranging from 12 to 14 months [4, 5]. Different factors are involved in GBM aggressiveness and poor prognosis, such as rapid cell proliferation, resistance to drug-induced apoptosis and enhanced invasiveness. Nevertheless, a small subset of patients presents a better outcome, surviving longer than 14 months: these patients are termed long-term survivors (LTS) [4]. The molecular events associated with the LTS phenotype are not well elucidated. Better understanding of these events would be critical for the development of early detection methods, identification of new biomarkers, and improved therapeutic approaches.

MicroRNAs (miRNAs or miRs) are a class of evolutionary conserved small non-coding RNAs that have great impact on a wide spectrum of biological processes. miRNAs act by affecting gene expression at the post-transcriptional level [6]. Many studies have demonstrated a pivotal role for miRNAs in tumorigenesis, acting both as oncogenes or tumor suppressors [7–10]. During tumor initiation and progression, miRNAs may modulate proliferation, angiogenesis, invasion and survival [11]. Deregulation of miRNA expression has been found in many human cancers, including GBM [12–15].

*NRAS* is a member of the *RAS* oncogene family (which comprises *KRAS*, *HRAS* and *NRAS*); they encode small GTPases involved in cellular signal transduction. RAS is activated by a complex signal cascade and, in turn, triggers downstream signaling pathways such as the mitogen-activate protein kinases (MAPKs) pathway and the phosphatidylinositol 3-kinase (PI3K)/AKT pathway, to modulate cell growth and survival [16]. Various studies have demonstrated recurrent aberrant *NRAS* activation in GBM [17]. Recently, several miRNAs–such as miR-181d, let-7 and miR-143–have been reported to suppress *RAS* expression, and thus act as tumor suppressors; this suggests that the dysregulation of miRNAs targeting *NRAS* may have an important role in carcinogenesis [8, 18–20].

For the present study, we investigated differential miRNA expression in long- and short-term GBM survivors. We identified miR-340 as a novel tumor suppressor miRNA that is up-regulated in LTS patients and predictive of better prognosis. Furthermore, we describe the oncosuppressive mechanisms induced by this miRNA: its ability to directly target *NRAS*, and thus silence downstream pathways, contributed in the blunting of the tumorigenic behavior of GBM cells.

## RESULTS

### miR-340 expression correlates with survival in GBM patients

To identify miRNAs de-regulated in long- vs short-term GBM survivors, we profiled the miRNA signatures of primary GBM tissue harvested from 3 LTS and 3 STS patients. The analysis was performed with a microarray chip containing 1150 miRNA probes, including 326 human and 249 mouse miRNAs, spotted in duplicate. Data obtained indicated that seven miRNAs (namely miR-193b, -340, -19b, -20a-b, -219-5p, -137 and -129-3p) were significantly de-regulated (> 1.5-fold change) in LTS vs STS GBM patients (Supplementary Table 1). We decided to focus our attention on miR-340, because we and others have already demonstrated the oncosuppressive role of this miRNA in different human tumors [21–27]. Accordingly with microarray data, qRT-PCR for miR-340 confirmed that it was up-regulated in LTS patients (Supplementary Figure 1A).

**Figure 1:**
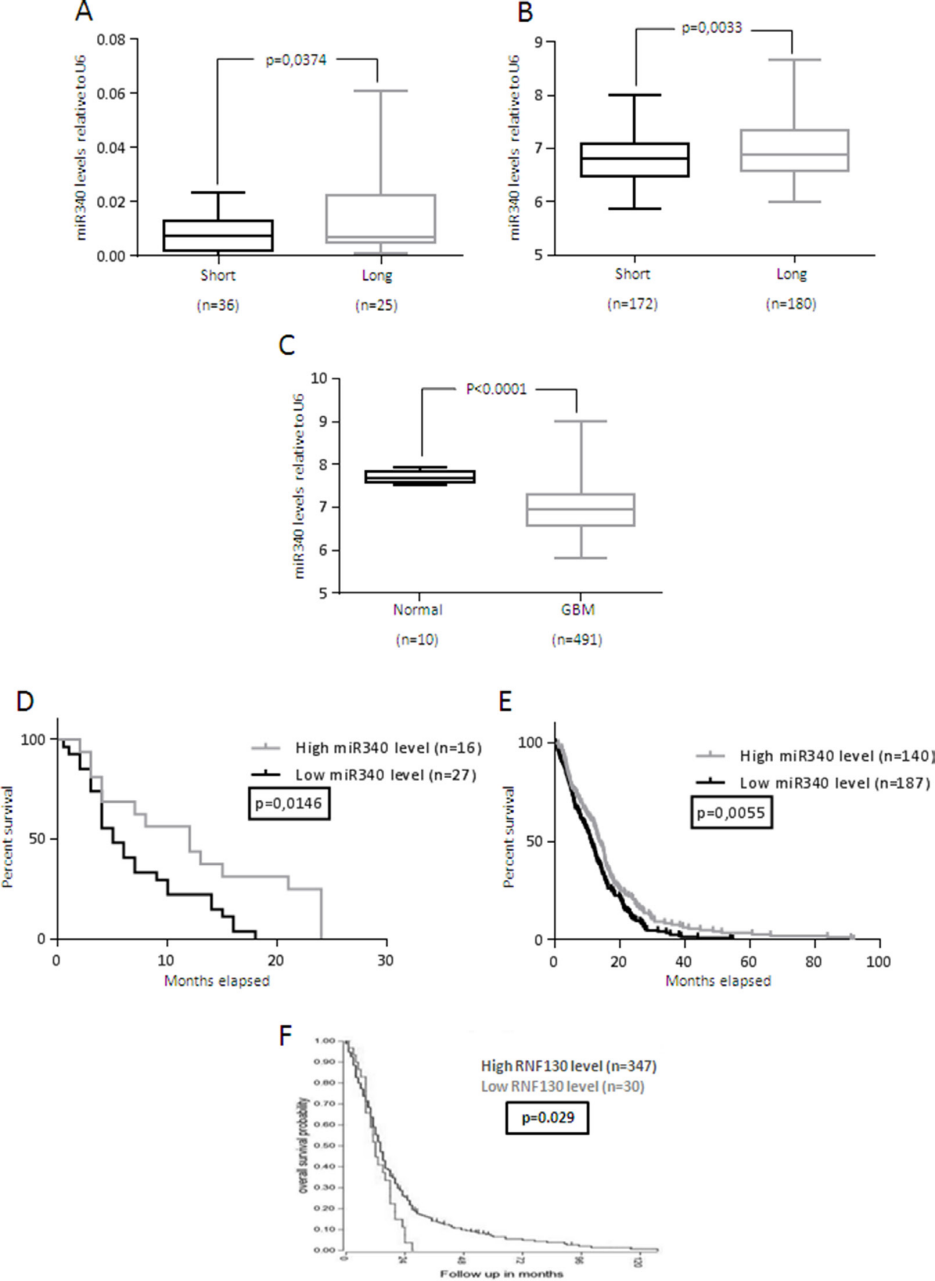
miR-340 is down-regulated in GBM and correlates with GBM prognosis miR-340 expression was evaluated using three independent patient cohorts (**A**) FFPE tissue from 36 LTS and 25 STS GBM patients; (**B**) 180 LTS and 172 STS GBM patients from TCGA database; (**C**) 10 normal brain specimens and 491 GBM tissues from TCGA database. A significant increase in miR-340 expression was identified between LTS vs STS in both cohorts and in normal brain vs GBM tissue. miR-340 expression was assessed by Real-Time PCR and normalized against U6. An arbitrary cut-off of 12 months was used to divide LTS and STS patients. Statistical significance was calculated using Student's *t*-test. *P* < 0.05 was considered significant. (**D**, **E**), Kaplan-Meier survival curve analysis of the correlation between miR-340 and overall survival of: (D) the FFPE tissues from 16 highly and 27 poorly miR-340-expressing glioblastoma patients; (E) 140 highly and 187 poorly miR-340-expressing glioblastoma patients collected from TCGA database. High miR-340 expression predicted a better prognosis in both cohorts. The patients were assigned to the high or low miR-340-expressing group using the media as a threshold. *P* was calculated using Log-Rank test. *P* < 0.05 was considered significant. (**F**) Kaplan-Meier survival curve analysis of the correlation between RNF-130 and overall survival of 347 highly and 30 poorly *RNF-130*-expressing glioblastoma patients from the R2.aml database. High RNF-130 expression predicted a better prognosis. *P* was calculated using Log-Rank test. *P* < 0.05 was considered significant.

We then analyzed miR-340 expression in a larger cohort of glioblastoma patients (*n* = 61), as well as in data collected from TCGA database (491 glioblastomas and 10 normal brain samples). As expected, miR-340 expression was significantly decreased in STS compared to LTS (*p* < 0.05; Figure 1A, 1B), and in GBM compared to normal brain (*p* < 0.001; Figure 1C). Furthermore, Log-Rank analysis of two different cohorts of GBM patients (43 GBM patients from our hospital and 327 from TCGA) indicated that patients with higher levels of miR-340 had longer overall survival, suggestive of a prognostic role of miR-340 (*p* < 0.05; *p* < 0.01). The Kaplan-Meier curves of the patient cohorts are given in Figure 1D–1E. Interestingly, higher levels of RNF130, the host gene of miR-340, was also predictive of a better prognosis in GBM patients (*p* < 0.05; fig 1f, data from R2.aml database). Finally, we found that miR-340 expression did not correlate with different glioma tumor stages (Supplementary Figure 1B) and with MGMT methylation status (Supplementary Figure 1C).

### *NRAS* mRNA is a direct target of miR-340

**Figure 2:**
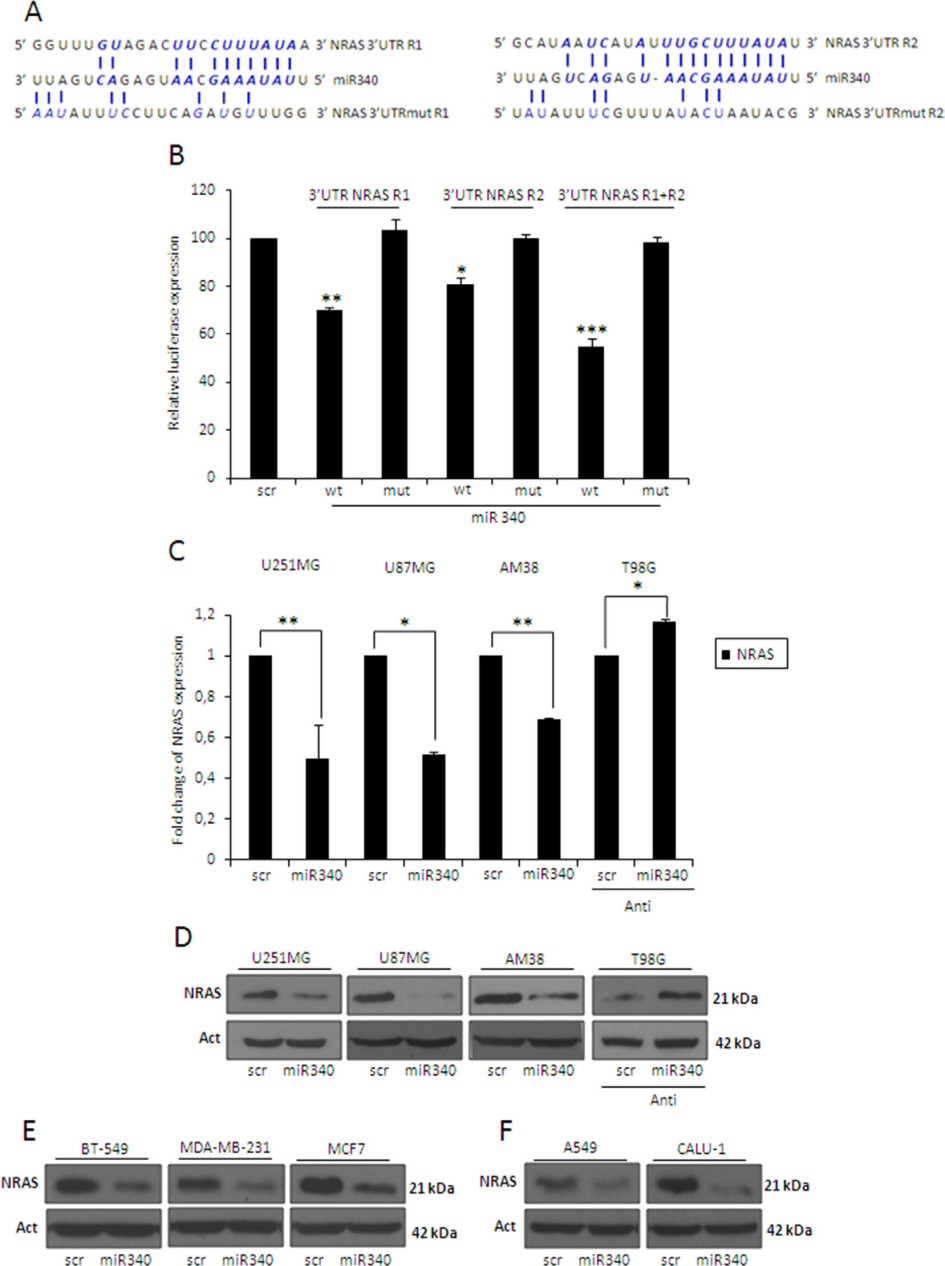
miR-340 targets *NRAS* (**A**) Predicted miR-340 binding sites on 2 sites on *NRAS*-3′UTR (3′UTR *NRAS* R1 and 3′UTR *NRAS* R2) found with the MIRANDA algorithm (www.microrna.org) and the designed mutant sequences (3′UTR NRAS R1mut and 3′UTR *NRAS* R2mut). (**B**) *NRAS* luciferase constructs containing wild-type or mutated *NRAS*-3′UTR-R1 or -R2, were co-transfected alone or in combination with miR-340 or a scrambled miRNA in A549 cells. Luciferase activity was measured 24 h after transfection. Reporter activities of cells co-transfected with the scrambled miRNA sequence have been arbitrarily set as 100. The results were obtained from three independent experiments and are presented as mean ± SD. In (B) *P* was calculated using ANOVA and adjusted for multiple comparisons with Bonferroni's *post hoc* testing. *p < 0.05; **p < 0.01; ***p < 0.001 (over Scr). Glioblastoma cell lines (U251MG, U87MG and AM38), breast cancer cell lines (BT-549, MDA-MB-231 and MCF7), and NSCLC cell lines (A549 and Calu-1) were transfected with a scrambled miRNA sequence and miR-340, or with a scrambled anti-miR-340 sequence and anti-miR-340 in T98G cells for 72 h. Real-time PCR (**C**) and Western blotting (**D–F**) were performed to analyze NRAS mRNA and protein levels. Western blot analyses are from representative experiments. Actin was used as loading control. The experiments were repeated at least three times. In (C) the results are presented as mean ± SD. *P* was calculated using Student's *t*-test. *p < 0.05; **p < 0.01.

To identify possible miR-340 targets involved in the LTS phenotype, we parsed bioinformatics databases (Targetscan, Miranda, Pictar). We found the presence of two distinct putative miR-340 binding sites on the 3′UTR of *NRAS* mRNA (Figure 2A). To assess if miR-340 directly bound to these two putative regions, we cloned them individually downstream of a luciferase reporter gene in the pGL3 vector. A549 NSCLC cells (expressing low endogenous level of miR-340) [25] were co-transfected with the reporter plasmids singularly or in combination, in the presence of either miR-340 or a control miRNA (scrambled). Luciferase activity of both reporters was repressed by the addition of miR-340 (Figure 2B); moreover, the effect was greater in cells co-transfected with both reporters, indicating that the two *NRAS* 3′UTR sites acted synergistically. Luciferase activity was not affected by miR-340 overexpression in the presence of mutant constructs, in which the seed sequences were cloned inversely (Figure 2A–2B).

To establish a causative effect between miR-340 and *NRAS*, we transfected different GBM cell lines (U251MG, U87MG and AM38 cells) with miR-340 and analyzed NRAS levels with qRT-PCR and Western blotting. We chose these three cell lines because they express low levels of miR-340, as assessed by qRT-PCR on a panel of 11 different GBM cell lines (Supplementary Figure 1D). We found a consistent, strong decrease in *NRAS* mRNA and protein in all cell lines transfected (Figure 2C–2D). In contrast, anti-miR-340 induced an increase in *NRAS* levels in T98G cells (Figure 2C–2D). Interestingly, miR-340 overexpression was also able to decrease NRAS protein levels in three different breast cancer cell lines (BT-549, MDA-MB-231, MCF7; Figure 2E) and two NSCLC cell lines (A549, Calu-1; Figure 2F). Furthermore, to test the ability of miR-340 to specifically target *NRAS* mRNA, we verified that both sequences binding this miRNA were not conserved in *KRAS* or *HRAS* 3′UTRs (data not shown); coherently, miR-340 overexpression did not decrease *KRAS* or *HRAS* mRNA levels in GBM cells (Supplementary Figure 2A–2B).

### Effects of miR-340 in GBM cell lines

**Figure 3:**
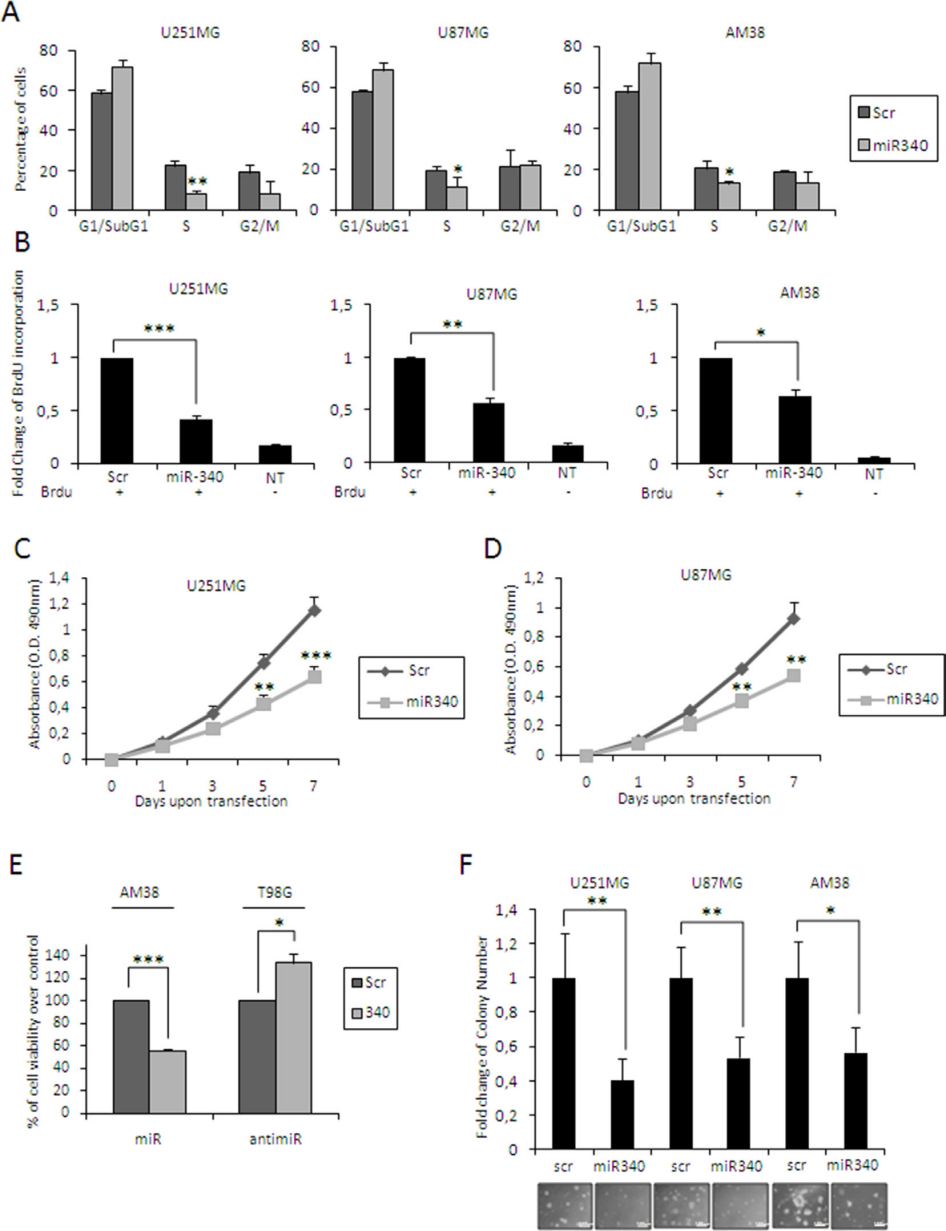
miR-340's effects in glioblastoma cells Glioblastoma cell lines (U251MG, U87MG and AM38) were transfected with miR-340 or with a control scrambled miRNA sequence. We then analyzed: (**A**) cell cycle by flow cytometry after propidium iodide staining 72 h after transfection; (**B**) cell proliferation by BrdU incorporation assay, 3 days after transfection; (**C**–**E**) cell proliferation by MTT assay, 1, 3, 5 and 7 days after transfection (3 days for AM38); (**F**) anchorage-independent cell growth by Soft Agar assay, 14 days after transfection. (E) T98G cells were transfected with anti-miR-340 or a control with scrambled anti-miRNA sequence and cell proliferation analyzed with MTT 3 days after transfection. miR-340 overexpression blocked cell cycle and decreased cell proliferation and anchorage-independent cell growth. The data are representative of three independent experiments. Data are mean values ± SD from three independent experiments. *P* was calculated using Student's *t*-test. *p < 0.05; **p < 0.01; ***p < 0.001.

We investigated the tumor suppressive role of miR-340 in different GBM cell lines (U251MG, U87MG and AM38 cells) transfected with either miR-340 or a control miRNA. We analyzed the effects of miR-340 on cell cycle, cell proliferation and soft agar growth. miR-340 transfection induced a significant S-phase block, as assessed by FACS analysis after PI staining (Figure 3A, plots for qualitative data analysis are reported in Supplementary Figure 3A), and a significant decrease in cell proliferation, as assessed by BrdU incorporation (Figure 3B; plots for qualitative data analysis are reported in Supplementary Figure 4A) and MTT assay (Figure 3C–3E). In contrast, expression of anti-miR-340 in T98G cells increased cell proliferation 72 h after transfection (Figure 3E). We next investigated whether miR-340 expression had an impact on anchorage-independent cell growth with a soft agar assay. We found that miR-340 induced a reduction in colony formation in U251MG, U87MG and AM38 cells (Figure 3F). These results clearly demonstrate that miR-340 acts as tumor suppressor in GBM by blunting cell cycle, proliferation and anchorage-independent cell growth.

### miR-340 sensitizes GBM cells to TMZ

**Figure 4:**
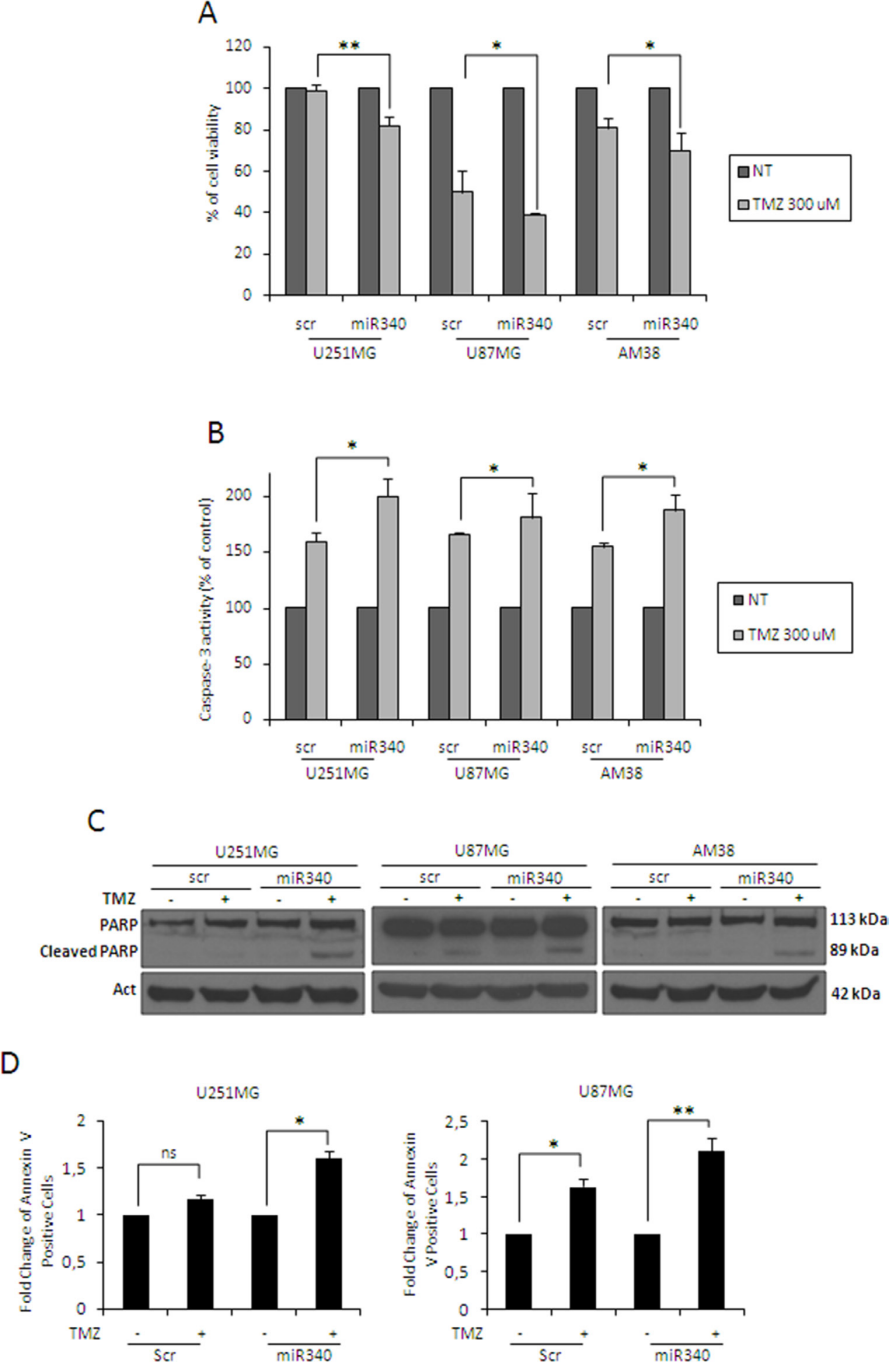
miR-340 increases TMZ sensitivity in glioblastoma cells Glioblastoma cell lines (U251MG, U87MG and AM38) were transfected with a scrambled miRNA sequence or miR-340 for 24 h, and then treated with 300 μM TMZ for 24 h. Cell death and apoptosis were analyzed respectively with the MTT assay (**A**) and caspase assay (**B**), western blot analysis for PARP (**C**), and Annexin V assay (**D**). miR-340 overexpression promoted TMZ-induced apoptosis. Data are mean values ± SD of three independent experiments. *P* was calculated using Student's *t*-test. *p < 0.05; **p < 0.01.

Because adjuvant chemotherapy with temozolomide is limited by the action of O-6-methylguanine-DNA methyltransferase (MGMT), a contributing factor for very poor survival in GBM patients [28], we investigated a possible role of miR-340 in TMZ sensitivity. MTT and Caspase 3/7 assays showed that miR-340 induced an increase in sensitivity to TMZ in all the cells analyzed (Figure 4A, 4B). Coherently, miR-340 overexpression determined an increase of apoptosis in GBM cells, as assessed by PARP cleavage (Figure 4C) as well as Annexin V assay (Figure 4D, plots for qualitative data analysis are reported in Supplementary Figure 5A). These results suggest that miR-340 expression contributes to the establishment of a LTS phenotype by enhancing the response of GBM patients to alkylating drugs.

### *NRAS*: a key target molecule of miR-340-mediated effects

**Figure 5:**
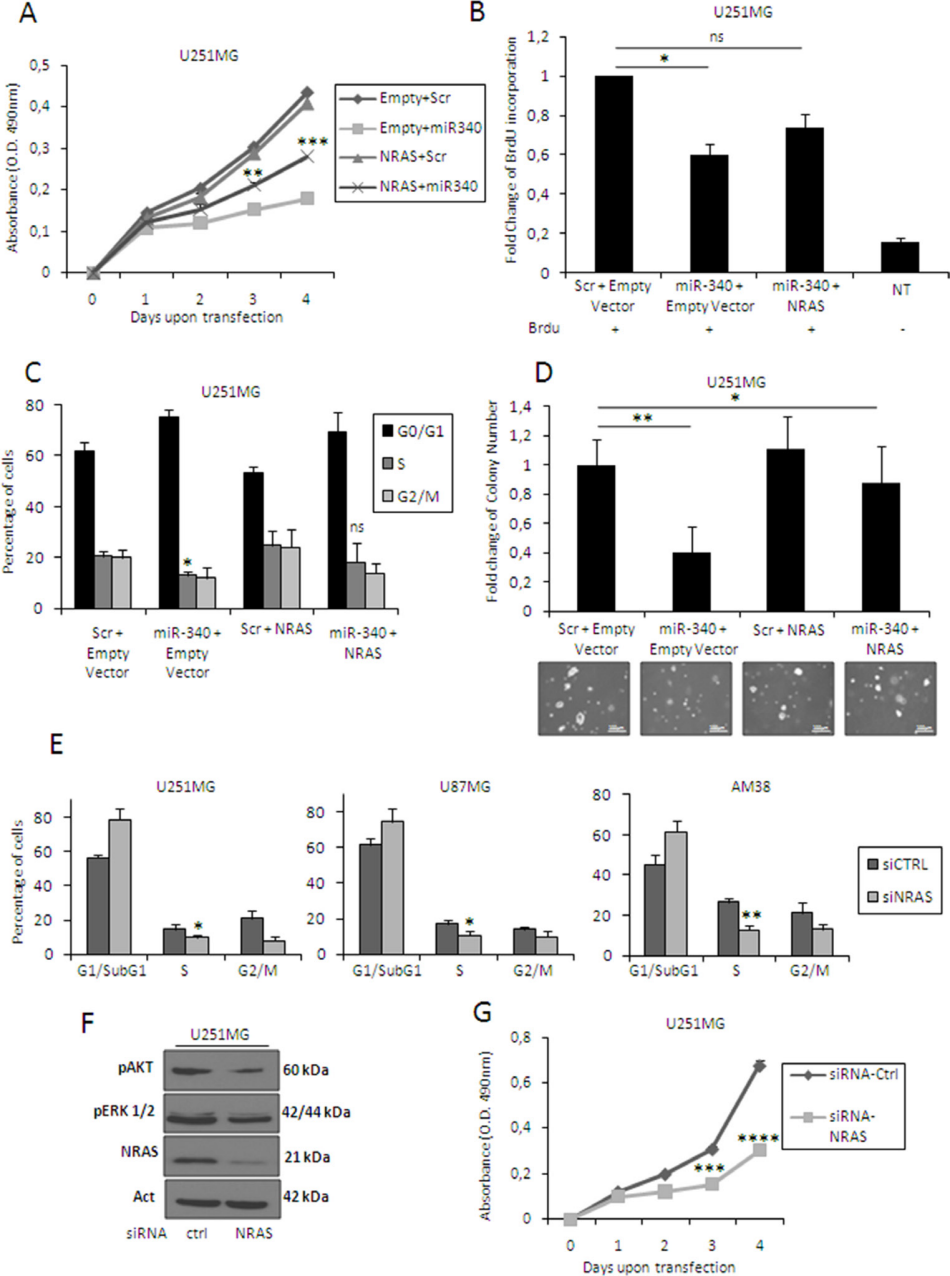
*NRAS* mediates the effects of miR-340 on cell cycle, proliferation and anchorage-independent cell growth U251MG cells were co-transfected with miR-340 and either a vector carrying *NRAS* lacking 3′UTR or a control. Exogenous *NRAS* expression partially counteracted the effects of miR-340 on proliferation (**A, B**), cell cycle (**C**), and anchorage-independent growth (**D**). Moreover, NRAS knock-down reproduced the effects of miR-340 transfection in GBM cells. U251MG, U87MG, and AM38 cells were transfected with a control siRNA or with a specific siRNA targeting *NRAS*. *NRAS* silencing mimicked the effects of miR-340 on cell cycle (**E**) decreased the phosphorylation of AKT and ERK (**F**) and reproduced the block of proliferation induced by miR-340 transfection (**G**). The experiments were repeated at least three times. Presented data are mean values ± SD of three independent experiments. Western blot analyses are representative experiments. Actin was used as the loading control. In (A–D) *P* was calculated using ANOVA and adjusted for multiple comparison with Bonferroni's *post hoc* testing (over Empty + Scr); in (E, G) *P* was calculated using Student's *t*-test. *p < 0.05; **p <0.01; ***p < 0.001; ****p < 0.0001.

*NRAS* is a key oncogene deregulated in many human cancers [16, 17, 29–31]. To demonstrate a causative link between miR-340 and *NRAS*, we performed rescue experiments, transfecting U251MG cells with miR-340 and with a construct expressing *NRAS* mRNA lacking the 3′UTR. Levels of transfected NRAS were detected by Western blotting (data not shown). Interestingly, expression of this *NRAS* counteracted the effects of miR-340 overexpression on proliferation (Figure 5A–5B; plots for qualitative data analysis are reported in Supplementary Figure 4B), cell cycle (Figure 5C; plots for qualitative data analysis are reported in Supplementary Figure 3B) and anchorage-independent cell growth (Figure 5D). Coherently, *NRAS* knock-down with a specific *NRAS* siRNA mimicked the effects of miR-340 overexpression, blocking cell cycle (Figure 5E; plots for qualitative data analysis are reported in Supplementary Figure 3C), decreasing the phosphorylation of ERK and AKT kinases (Figure 5F), and reducing proliferation (Figure 5G).

We then analyzed *NRAS* expression in a cohort of 39 GBM patients: we found that *NRAS* was down-regulated in LTS vs STS patients (*p* < 0.05, Supplementary Figure 6A). Moreover, *NRAS* expression was higher in 542 GBM samples compared to 10 normal brain specimens (data collected from the oncomine database; *p* < 0.001; Supplementary Figure 6B). Furthermore, Log-Rank analysis of 28 GBM patients showed that those with higher levels of *NRAS* had shorter overall survival (Supplementary Figure 6C). This finding was confirmed by data from the R2.aml database (504 tissues; *p* < 0.05; Supplementary Figure 6D). In conclusion, the anti-tumoral effects of miR-340 seem, at least in part, to be mediated by its targeting of *NRAS*.

Recently, miR-340 was reported to directly target two important oncogenes, *SKP2* and *ROCK1*, respectively in lung cancer and in osteosarcoma [22, 25, 26]. We found that miR-340 decreased the expression of both these oncogenes in three different GBM cell lines, suggesting that miR-340 may affect GBM tumorigenesis also by targeting other mRNAs (Supplementary Figure 7A).

### miR-340 blocks cell cycle and cell proliferation via inhibition of signaling pathways downstream from NRAS

**Figure 6:**
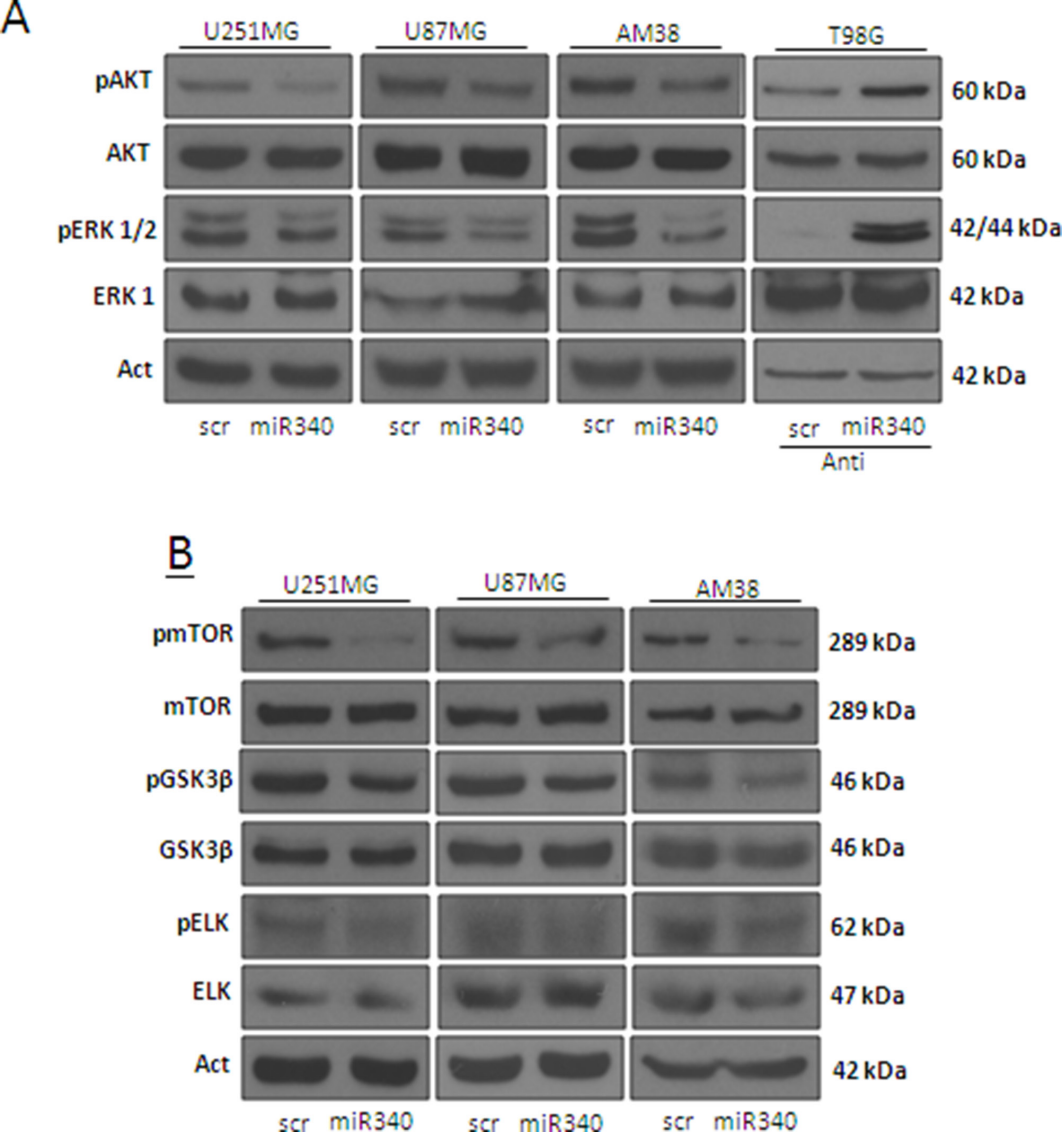
miR-340 overexpression decrease the activation of molecular pathways downstream NRAS Glioblastoma cell lines (U251MG, U87MG and AM38) were transfected with a scrambled miRNA sequence or miR-340, or with a scrambled anti miRNA sequence or anti miR-340, in T98G cells, for 72 h. Western blotting was performed to analyze pAKT and pERK1/2 (**A**) and pmTOR, pGSK3β, and pELK protein levels (**B**) Western blot analyses are representative experiments. Actin was used as the loading control. The experiments were repeated at least three times.

AKT and ERK1/2 pathways act as major downstream signals of RAS, promoting multiple oncogenic features of RAS, such as cell proliferation and apoptosis resistance. We assessed the levels of phosphorylated (activated) forms of AKT and ERK1/2 (p-AKT and p-ERK1/2) by Western blotting in glioblastoma cells transfected with miR-340 or a control scrambled sequence. miR-340 drastically reduced p-AKT and p-ERK1/2 levels in U251MG, U87MG and AM38 cells (Figure 6A). In contrast, transfection with anti-miR-340 induced an increase in p-AKT and p-ERK1/2 in T98G cells (Figure 6A). Interestingly, performing a more comprehensive analysis of the AKT and ERK pathways, we found that miR-340 overexpression decreased the phosphorylation of other kinases of both AKT (mTOR, GSK3β), and ERK pathway (ELK) (Figure 6B).

**Figure 7:**
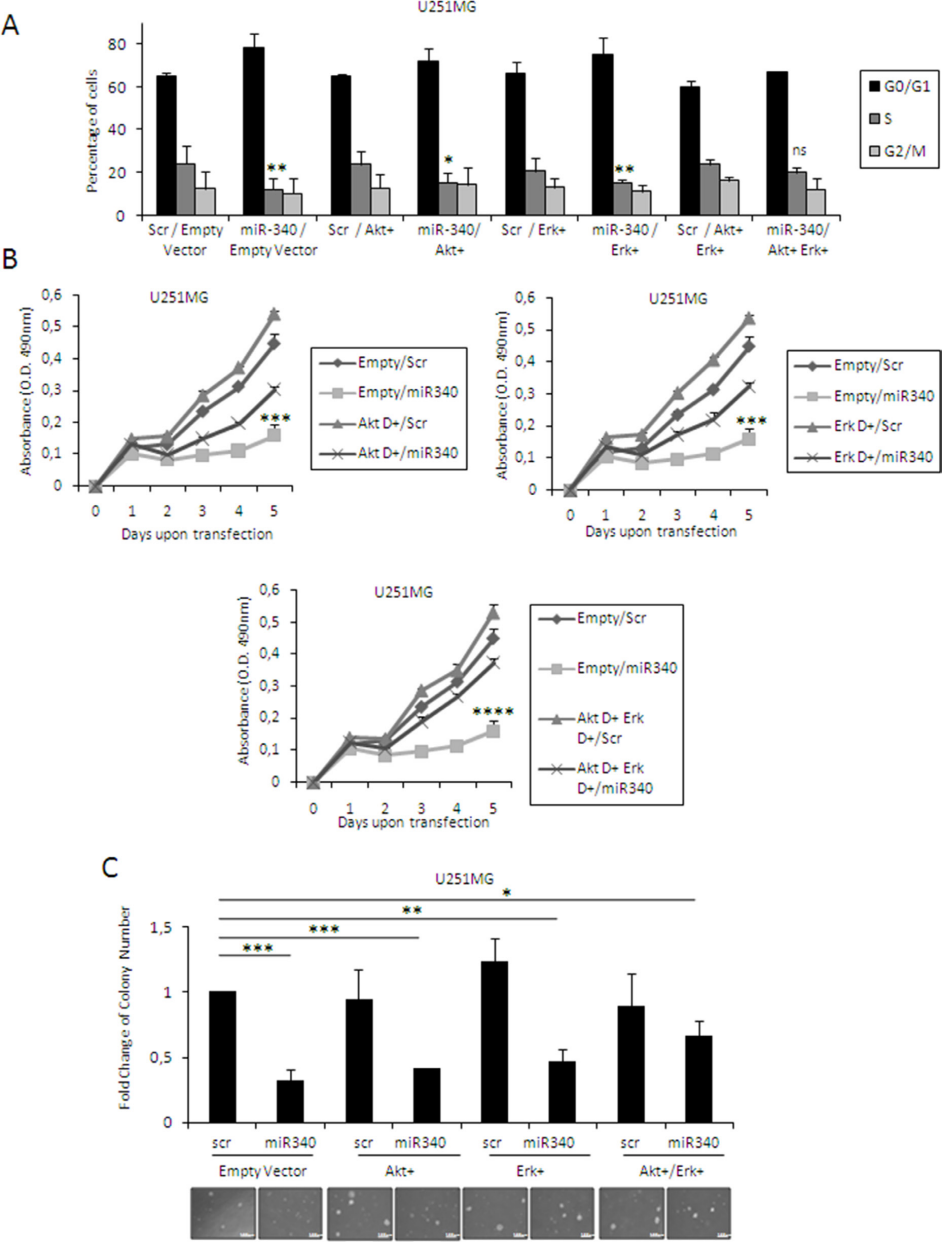
Molecular pathways downstream from NRAS mediate the effects of miR-340 GBM cells U251MG glioblastoma cells were co-transfected with miR-340 and dominant active mutants constructs of ERK and AKT, alone or in combination, or with control vector. Exogenous dominant active expression of both ERKs and AKT was able to partially counteract the effects of miR-340 on cell cycle (**A**) proliferation (**B**) and anchorage independent cell growth (**C**) The experiments were repeated at least three times. Presented data are mean values ± SD of three independent experiments. In (A) *P* was calculated using Student's *t*-test. In (B, C) *P* was calculated using ANOVA and adjusted for multiple comparisons with the Bonferroni's *post hoc* testing. *p < 0.05; **p < 0.01; ***p < 0.001; ****p < 0.0001 (over Empty + Scr).

Next, we wondered whether miR-340-mediated blockade of cell cycle and cell proliferation was mediated by inhibition of AKT and ERK1/2 signaling pathways downstream of NRAS. To this aim, we transfected U251MG cells with miR-340 and two constructs expressing constitutively active forms of AKT (AKT^+^) and ERK1 (ERK^+^) for 48 h, either alone or in combination. Levels of transfected AKT^+^ and ERK^+^ were assessed by Western blotting (data not shown). AKT^+^ and ERK^+^ were individually able to partially counter the effects of miR-340 on cell cycle (Figure 7A; plots for qualitative data analysis are reported in Supplementary Figure 3D), proliferation (Figure 7B), and anchorage independent cell growth (Figure 7C); the effects were greater when AKT^+^ and ERK^+^ were co-transfected (Figure 7A–7C). These results further support the notion that miR-340 acts as a tumor suppressor in GBM by targeting NRAS and, hence, blunting downstream AKT and ERK1/2 pathways.

### Overexpression of miR-340 inhibits GBM growth *in vivo*

**Figure 8:**
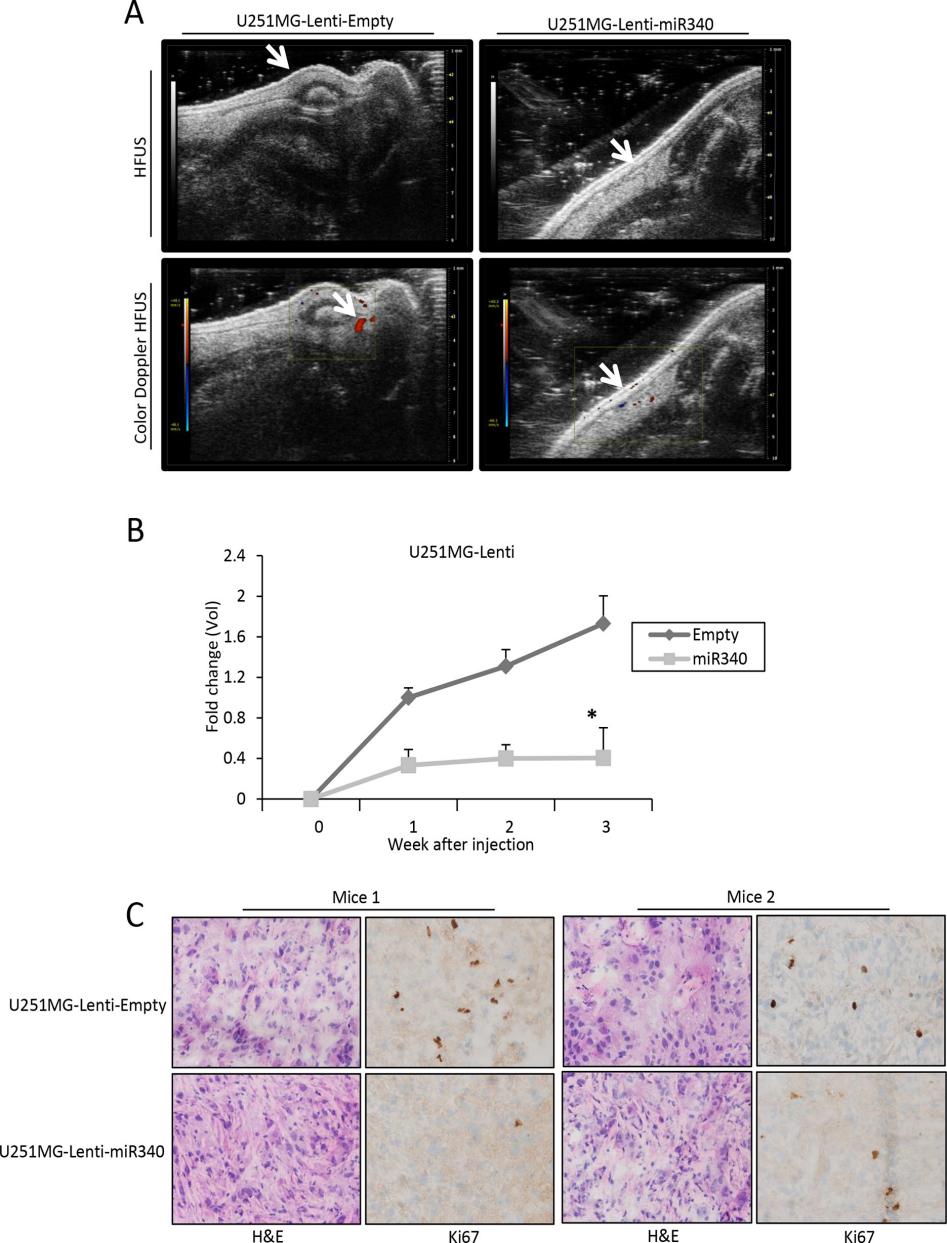
miR-340 inhibits the growth of glioblastoma xenografts *in vivo* U251MG cells stably transduced with a lentiviral vector encoding miR-340 or with control particles were subcutaneously injected into the left flank of CD1 nude mice (*n* = 6 per group). Tumor volume and vessel formation were measured weekly by HFUS and color-doppler HFUS (Vevo 2100 with a 40MHz probe) for three weeks. Data in (**A**) and (**B**) show that treatment with miR-340 reduced glioblastoma-derived xenograft growth and vessel formation. In (**B**) *P* was calculated using Student's *t*-test. *p < 0.05. (**C**) Nuclear localization of KI67 staining and HE staining of xenograft mice injected with U251MG-Lenti-Empty expressing cells and U251MG-Lenti-miR340 overexpressing cells. A representative area of each tissue is represented. Two mice for each experimental point are represented. Magnification 40x (Figure 8C).

To analyze the possible role of miR-340 in GBM tumorigenesis, we assessed the effects of miR-340 overexpression on tumor growth *in vivo*. To this end, we stably infected U251MG cells with a lentiviral construct expressing either miR-340 or control sequence (Supplementary Figure 8A–8B). In accordance with previous data, miR-340 stable infected cells showed a reduction of proliferation and a block of cell cycle (Supplementary Figure 8C–8D). These cells were subcutaneously injected into the left flank (2 × 10^6^ cells per flank) of CD1 nude mice (*n* = 6 animals per group). Tumor volume and vessel formation were measured weekly by HFUS and color-doppler HFUS for 3 weeks. Tumor volume and vessel formation generated by miR-340-expressing U251MG xenografts were significantly less compared to those of control xenografts (Figure 8A, 8B). Coherently, Ki67 staining of tumor sections indicated a decrease of cell proliferation in miR-340-expressing U251MG xenografts (Figure 8C).

## DISCUSSION

GBM is one of the most aggressive types of human cancer and the most lethal form in the brain. It is characterized by extremely bad prognosis, with a median survival rate of only 12 months from diagnosis [2]. Interestingly, a small subgroup of patients survives longer. Better understanding of the specific molecular features underlying this LTS phenotype may lead to improved diagnosis, prognosis, treatment and, ultimately, survival of GBM patients [4]. At present, several molecular markers have been correlated to GBM prognosis, but they need further validation before they can be used in the clinical setting [28]. However, despite great effort over the last years, the cellular and molecular features of LTS have not been properly elucidated.

In the last decade, microRNAs have been frequently found deregulated in different human cancers, acting both as oncogenes or tumor suppressors. miRNAs are involved in basic cellular functions, including proliferation, cell death, differentiation, metabolism and, importantly, tumorigenesis [7–11, 13]. In addition, these noncoding RNAs have the capacity to target tens to hundreds of genes simultaneously [6]. Thus, they are attractive candidates as prognostic biomarkers and therapeutic tools/targets in cancer. Several studies have demonstrated that the expression of miRNAs is deregulated in gliomas [12, 14, 15, 18] and may affect GBM tumorigenesis [32–34]. More importantly, several reports have established a direct link between miRNA signatures and GBM prognosis [35–38]. In the present study, we identify miR-340 as a tumor-suppressive miRNA in GBM. Our experimental data, together with that obtained from TCGA database, show that miR-340 expression is significantly higher in LTS compared to STS patients, and in normal brain compared to GBM. Furthermore, statistical analysis revealed that patients with higher miR-340 expression had a longer survival rate and, thus, a better prognosis. Taken together, these data suggest that higher expression of miR-340 is a significant predictor of good prognosis in GBM.

Our functional studies show that overexpression of miR-340 in GBM cells determines a significant block of cell cycle, inhibition of cell proliferation, decrease of anchorage-independent cell growth, and an increase in sensitivity to TMZ. Furthermore, miR-340 directly binds to two different sites on the 3′UTR of *NRAS* mRNA to strongly inhibit NRAS protein expression. The RAS protein family consists of 4 highly homologous enzymes (NRAS, HRAS, KRAS4A and KRAS4B) that act as signaling molecules for the regulation of cell fate: they couple receptor activation to downstream effector pathways controlling different cellular responses, such as proliferation, resistance to therapy and survival [16]. Activating mutations of RAS proteins are common in human cancers, especially in hematopoietic cancers, colorectal cancer and melanoma [29–31]. In glioblastoma, *NRAS* activation could be caused by a direct mutation (5%) or by other alterations, such as amplification, overexpression of growth factor receptor or aberrations in other *RAS* pathway genes [17]. Recently, several miRNAs–including miR-181d, let-7 and miR-143–were reported to suppress RAS expression and function as tumor suppressors, suggesting that miRNAs targeting *RAS* may have an important role in carcinogenesis [18–20]. Our findings indicate that miR-340's targeting of *NRAS* leads to a decrease in the activation of downstream pathways (namely AKT and ERKs). Experiments using dominant positive mutants of AKT and ERKs clearly show that both these pathways are down-regulated by a miR-340-mediated effect in GBM. Thus, we have identified a new link between miR-340 and NRAS, a novel player in GBM tumorigenesis.

miR-340 has been shown to have a tumor-suppressive activity also in other types of cancer. Wu et al. reported that miR-340 suppressed cell growth in breast cancer [21]; in osteosarcoma, miR-340 was found down-regulated compared to normal tissue, and *ROCK-1* was identified as a miR-340 target [22, 26]; Poenitzsch et al. demonstrated for the first time the pleiotropic regulation of the RAS-RAF-MAPKs pathway by miR-340 in melanoma, which resulted in a strong tumor-suppressive activity [24]; Fernandez et al. characterized the tumor-suppressive activity of miR-340 in lung cancer, where it mediated cell growth inhibition and apoptosis activation via the accumulation of p27 [25]; Yamashita et al. reported that miR-340 suppressed the stem-like cell function of glioma-initiating cells *in vitro* and in nude mouse brain–their findings indicated that miR-340 acted as a tumor suppressor in glioma-initiating cells, particularly affecting gliomagenesis and extensive tumor invasion [23]. Recently Huang et al. reported that miR-340 was able to inhibit glioblastoma tumorigenesis [27].

In the present study, we show that lentiviral vector-mediated overexpression of miR-340 in GBM cells inhibits cell growth in nude mice, suggestive of its possible therapeutic use.

In conclusion, we have observed for the first time a direct link between miR-340 expression and survival in GBM, demonstrating that miR-340 has a powerful oncosuppressive effect *in vitro* and *in vivo*. The mechanism is mediated, at least in part, by the targeting of *NRAS* and the consequential blunting of downstream ERKs and AKT pathways. We propose miR-340 as a novel biomarker for GBM prognosis, as well as a new therapeutic agent for the amelioration of GBM patient survival.

## MATERIALS AND METHODS

### Cells and tissue specimens

Glioblastoma cell lines T98G, U87MG, LN229 and LN18, breast cancer cell lines BT549, MDA-MB-231, MCF7, and NSCLC cell lines A549 and CALU-1 were obtained from American Type Culture Collection (ATCC), (LG Standards, Milan, Italy); U251MG, LN428, LN308, SF767 and A172 were kindly donated by Frank Furnari (La Jolla University, San Diego, CA, USA). U87MG, U251MG, T98G, AM38, A172, LN319, LN308, LN428, SF767, MCF7 and Calu-1 were grown in Dulbecco's modified eagle's Medium, LN18 and LN229 in Advanced Dulbecco's modified eagle's Medium, and BT-549, MDA-MB-231 and A549 in RPMI Medium. Media were supplemented with 10% heat-inactivated fetal bovine serum (FBS) −5% FBS for LN229 and LN18–2 mM L-glutamine and 100 U/ml penicillin/streptomycin. All media and supplements were from Sigma Aldrich (Milan, Italy).

A total of 61 formalin-fixed, paraffin-embedded (FFPE) tissue samples were collected from the archives of the Department of Pathology, University Hospital of Kuopio, Finland. Among the 61 samples, survival information for 43 cases was available. Permission to use the material was obtained from the National Supervisory Authority for Welfare and Health of Finland, and the study was accepted by the ethical committee of the Northern Savo Hospital District, Kuopio, Finland. Informed consent was obtained from each subject or subject's guardian.

### TCGA data analysis

The collection of data from The Cancer Genome Atlas (TCGA) platform was compliant with laws and regulations for the protection of human subjects, and necessary ethical approvals were obtained. Analysis of all data was done using GraphPad Prism 6 (San Diego, CA, USA). For differential expression analysis and determination of the effect of miR-340 and *NRAS* on patient's survival, we downloaded Agilent 8 × 15 miRNA expression (level 2) and HT_HG-U133A (level 3) along with clinical information from the TCGA database in April 2014.

### Cell transfection

For transient overexpression of miRNAs and siRNAs, cells at 50% confluence were transfected using Oligofectamine (Invitrogen, Milan, Italy) and 100 nM of pre-miR-340, scrambled miRNA, anti-miR-340, scrambled anti-miRNA (Ambion^®^, Life Technologies), siNRAS or a siRNA control (Santa Cruz Biotechnologies, MA, USA). For transient overexpression of 4 μg of pcDNA3-NRAS, pcDNA3-AKT^+^, pcDNA3-ERK^+^ or pcDNA3, cells were transfected using X-tremeGENE 9 DNA Transfection Reagent (Roche, Milan, Italy). Temozolomide (TMZ) was purchased from Sigma Aldrich (Milan, Italy).

### RNA extraction and real-time PCR

*Cell culture:* Total RNA (microRNA and mRNA) was extracted using Trizol (Invitrogen, Milan, Italy) according to the manufacturer's protocol. *Tissue specimens:* total RNA (miRNA and mRNA) from FFPE tissue specimens was extracted using RecoverAll Total Nucleic Acid isolation Kit (Ambion, Life Technologies, Milan, Italy) according to the manufacturer's protocol. Reverse transcription of total RNA was performed starting from equal amounts of total RNA/sample (500 ng) using miScript reverse Transcription Kit (Qiagen, Milan, Italy) for miRNA analysis, and with SuperScript^®^ III Reverse Transcriptase (Invitrogen, Milan, Italy) for mRNA analysis. Quantitative analysis of miR-340 and RNU6A (the latter as an internal reference) were performed by RT-PCR using specific primers (Qiagen, Milan, Italy) and miScript SYBR Green PCR Kit (Qiagen, Milan, Italy). RT-PCR was also used to assess the mRNAs of NRAS, KRAS, HRAS and β-actin (the latter as an internal reference), using iQTM SYBR Green Supermix (Bio-Rad, Milan, Italy). The primer sequences were: NRAS-Fw: 3′-CGCACTGACAATCCAGCTAA-5′; NRAS-Rv: 3′-TCGCCTGTCCTCATGTATTG-5′; K-RAS-Fw: 3′-ACTGGGGAGGGCTTTCTTTG-5′; K-RAS-Rv: 3′-GGCATCATCAACACCCTGTCT-5′; H-RAS: Fw: 3′-TATAAGCTGGTGGTGGTGGG-5′; H-RAS-Rv: 3′- TGATGGCAAACACACACAGG-5′; Act-FW: 5′-TGCGTGACATTAAGGAGAAG-3′; Act-Rv: 5′-GCTCGTAGCTCTTCTCCA-3′.

The reaction for detection of mRNAs was performed in this manner: 95°C for 5 min, 40 cycles of 95°C for 30 s, 60°C for 30 s and 72°C for 30 s. The reaction for detection of miRNAs was performed in this manner: 95°C for 15 min, 40 cycles of 94°C for 15 s, 55°C for 30 s and 70°C for 30 s. All reactions were run in triplicate. The threshold cycle (CT) was defined as the fractional cycle number at which the fluorescence passed the fixed threshold. For relative quantization, the 2^(−ΔΔCT)^ method was used. Experiments were carried out in triplicate for each data point, and data analysis was performed with Applied Biosystems'stepOnePlus™ Real-Time PCR System.

### miRNA expression microarray and data analysis

From each sample, 5 μg of total RNA (from 3 long- and 3 short-term GBM survivors) was reverse transcribed using biotin end-labeled random octamer oligonucleotide primer. Hybridization of biotin-labeled cDNA was performed on an Ohio State University custom miRNA microarray chip (OSU_CCC version 3.0), which contained 1150 miRNA probes, including 326 human and 249 mouse miRNA genes, spotted in duplicates. The hybridized chips were washed and processed to detect biotin-containing transcripts with a streptavidin-Alexa647 conjugate and scanned on an Axon 4000B microarray scanner (Axon Instruments, Sunnyvale, CA, USA). Raw data were normalized and analyzed with GENESPRING 7.2 software (zcomSilicon Genetics, Redwood City, CA, USA). Expression data were median-centered with the GENESPRING normalization option and with the BIOCONDUCTOR package (www.bioconductor.org) global median normalization tool, with similar results. Statistical comparisons were done with the GENESPRING ANOVA tool, predictive analysis of microarray and the significance analysis of microarray software (http://www.stat.stanford.edu/Btibs/SAM/index.html).

### Establishment of glioblastoma cells stably expressing miR-340

Lentiviral vectors encoding an expression cassette containing a puromycin resistance gene, the green fluorescent protein (GFP) gene and the miR-340 sequence under the hCMV promoter were purchased from GE Healthcare Dharmacon (Milan, Italy). U251MG cells were infected with the miR-340 lentiviral vector or with an empty, control vector (which lacked the miR-340 sequence) at a final concentration of 20 MOI. After culturing in selection media supplemented with puromycin, GFP was detected by fluorescence microscopy (original magnification 10x; scale bar 100 μm), and representative images were collected using the Leica Application Suite X (LAS X) software (Leica, Milan, Italy). Finally, puromycin-resistant, GFP-positive clones were picked.

### Protein isolation and Western blotting

Cells were lysed in JS buffer (50 mM HEPES, pH 7.5, containing 150 mM NaCl, 1% glycerol, 1% Triton X-100, 1.5 mM MgCl_2_, 5 mM EGTA, 1 mM Na_3_VO_4_, and 1X protease inhibitor cocktail). Protein concentration was determined with the Bradford assay (BioRad, Milan, Italy) using bovine serum albumin as the standard, and equal amounts of proteins were analyzed by SDS-PAGE (12% acrylamide). Gels were electroblotted into nitrocellulose membranes (G & E Healthcare, Milan, Italy). Membranes were blocked for 1 h with 5% non-fat dry milk in tris-buffered saline (TBS) containing 0.1% Tween-20, and incubated at 4°C overnight with the primary antibody. Detection was performed by peroxidase-conjugated secondary antibodies, using the enhanced chemiluminescence system (Thermo Euroclone, Milan, Italy). Primary antibodies used were: anti-NRAS, anti-ERK1, anti-SKP2, anti-ROCK1 (Santa Cruz Biotechnologies, MA, USA), anti-pP42/44, anti-pAKT, anti-AKT, anti-pmTOR, anti-mTOR, anti-pGSK3β, anti-GSK3β, anti-pELK, anti-ELK (Cell Signaling, Danvers, MA, USA), and anti-βactin (Sigma Aldrich, Milan, Italy).

### MTT assay

Cell vitality was evaluated with the CellTiter 96^®^ AQueous One Solution Cell Proliferation Assay (Promega, Madison, WI, USA), according to the manufacturer's protocol. The assay is based on reduction of 3-(4,5-dimethylthiazol-2-yl)-5-(3- carboxymethoxyphenyl)-2-(4-sulfophenyl)-2H-tetrazolium, inner salt (MTS) to a colored product that is measured spectrophotometrically. After 24 h from miRNA or siRNA transfection, cells (1 × 10^3^) were plated in 96-well plates in triplicate and incubated at 37°C in a 5% CO_2_ incubator. Metabolically active cells were detected by adding 20 μl of MTS to each well; after 30 min of incubation, the plates were analyzed on a Multilabel Counter (Bio-Rad, Richmond, VA, USA).

### BrdU incorporation assay

Cell proliferation was evaluated with the *In Situ* Cell Proliferation Kit, FLUOS (Sigma Aldrich, Milan, Italy), according to the manufacturer's protocol. The assay is based on the incorporation of BrdU only in actively proliferating cells. Cells were plated in p100 plates and transfected with miR-340 or scrambled sequence. After 72 h, proliferating cells were detected by adding BrdU to each plate; after 4 h of incubation, the cells were fixed and labeled with anti-BrdU antibody conjugated with fluorescein for 45 min. Then, BrdU incorporation were detected with a Becton Dickinson FACScan flow cytometer.

### Cell cycle analysis

Cell cycle was analyzed via propidium iodide (PI) incorporation in permeabilized cells by flow cytometry. The cells (5 × 10^4^) were washed in PBS and resuspended in 200 μl of a solution containing 0.1% sodium citrate, 0.1% triton X-100 and 50 μg/ml propidium 6 iodide (Sigma Aldrich, Milan, Italy). Following incubation at 4°C for 30 min in the dark, nuclei were analyzed with a Becton Dickinson FACScan flow cytometer. Cellular debris was excluded from analyses by raising the forward scatter threshold, and the DNA content of the nuclei was registered on a logarithmic scale. The percentage of elements in the hypodiploid region was calculated.

### Soft-agar assay

1 × 10^4^ cells were plated in 60mm dishes in a solution containing Dulbecco's modified Eagle's medium 2 × (Sigma, St Louis, MO, USA), TPB buffer (Difco, BD, Franklin Lakes, NJ, USA), and 1.25% Noble Agar (Difco, BD, Franklin Lakes, NJ, USA). Briefly, cells were harvested and counted, then a layer of 7 ml of Noble Agar solution was left to polymerize on the bottom of the dishes. Then cells were resuspended in 2 ml of same solution and plated. Cells were left to grow for 2 weeks in the incubator.

### Cell death quantification and caspase assay

Cells were transfected with miRNAs as described and were plated in 96-well plates in triplicate, treated, and incubated at 37°C in a 5% CO_2_ incubator. Temozolomide was used at a final concentration of 300 μM for 24 h. Cell viability was assessed using the CellTiter 96^®^ AQueous One Solution Cell Proliferation Assay (Promega, Madison, WI, USA), as described above. Apoptosis activation was analyzed with the Caspase-Glo^®^ 3/7 Assay System (Promega, Madison, WI, USA), as reported in the instruction manual. Briefly, cells were incubated with medium supplemented with caspase 3/7 reagent; luminescence was measured following incubation for 30 min at room temperature.

### Apoptosis assessment by Annexin V staining

GBM cells were transfected with miR-340 or scrambled sequence. After 48 h, cells were treated with 300 μM of TMZ for 24 h, harvested, washed twice with cold PBS, and stained with Annexin V-FITC Apoptosis Detection Kit 1 (BD Pharmingen, Milan, Italy). Briefly, cells were resuspended in 100 μL of 1 × binding buffer and 5 μL of Annexin V and then incubated for 15 min at room temperature. Apoptotic cells were analyzed by flow cytometry.

### Rescue experiments

To determine whether *NRAS* mediated the effect of miR-340, rescue experiments were performed in which the effects of miR-340 were measured in the setting of overexpression of a deletion mutant of *NRAS*, i.e., one lacking the 3′ untranslated region (UTR). Cells were transfected with miR-340 and with the mutant *NRAS* vector, using X-tremeGENE 9 DNA Transfection Reagent (Roche, Milan, Italy), as described. Growth and cell cycle were assessed as above.

### *In vivo* tumor formation

Five-week-old female CD1 nude mice (Charles River, Milan, Italy) were maintained in special pathogen free conditions for one week. The animal protocols used in this work were evaluated and approved by the Animal Use and Ethic Committee (OBA) of the Institute Ceinge, Biotecnologie Avanzate s.c.a.r.l. (Protocol 15/1/14_n 4). The animals protocols were performed in accordance with FELASA guidelines and the guidelines defined by the European Communities Council directive (2010/63/EU). The investigators adhere to widely accepted national standards. U251MG cells stably expressing either miR-340 or miR-Empty were injected subcutaneously into the left flank of the nude mice (2 × 10^6^ cells in 100 μl). Tumor size was assessed weekly with a Vevo 2100 equipment (FUJIFILM VisualSonics, Inc., Toronto, Ontario, Canada), an high-frequency ultrasound (HFUS) system mounting a 40 MHz probe, 1, 2, and 3 weeks after cells injection. The procedures were performed under general anesthesia with 2% isoflurane in 100% oxygen at 0.8 L/min. For each tumor, mediolateral, anteroposterior and craniocaudal diameters were measured. Tumor volume (TV) was calculated according to the formula V = (height × width × length × 3.16)/6.

### Immunohistochemical staining and evaluation

Xenograft fresh frozen tissue were embedded in OCT compound and were cut in sections of 5 μm thickness. Staining was performed with an automatic Benchmark XT staining machine (Ventana Medical Systems Inc., Tucson, AZ, USA) with an antihuman KI67 (Ventana) antibody according to manufacture procedure. KI67 nuclear staining intensity was evaluated by one expert pathologist. For H & E staining, 2.5 μm sections of all fixed samples were mounted on superfrost slides and performed using standard methodology.

### Luciferase assay

The two predicted regions on the 3′UTR of the human *NRAS* gene (R1 and R2) containing the putative miR-340 binding site were PCR amplified using the following primers: NRAS-R1-FW: 3′-GCTCTAGATGGCATCTGCTCTAGATTCATAAA-5′; NRAS-R1-Rv: 3′-GCTCTAGATGGCATCTGCTCTAGATTCATAAA-5′; NRAS-R2-FW: 3′-GCTCTAGACTATTTTAGTGGGCCCATGTT-5′; NRAS-R2-Rv: 3′-GCTCTAGACAAGAAGCAGAACGCACC-5′, and cloned downstream of the Renilla luciferase stop codon in a pGL3 control vector (Promega, Milan, Italy). An inverted sequence of the miRNA-binding sites was used as the negative control. A549 cells were transfected with miR-340 or a scrambled miRNA for 6 h. Then, the cells were co-transfected with 1.2 μg of 3′UTR NRAS-R1 or -R2 plasmids, or relative mutant constructs, plus 400 μg of a Renilla luciferase expression construct, pRL-TK (Promega, Milan, Italy), with Lipofectamine 2000 (Life Technologies, Milan, Italy). Cells were harvested 24 h post-transfection, and the luciferase activity assayed with Dual Luciferase Assay (Promega, Milan, Italy), according to the manufacturer's instructions. Three independent experiments were performed in triplicate.

### Statistical analysis

All experiments were repeated at least three times. Continuous variables are given as mean ± 1 standard deviation (SD). For two-groups comparison, Student's *t*-test was used to determine differences between mean values for normal distribution. Comparisons among more than two groups were determined by one-way ANOVA followed by Bonferroni's *post hoc* testing. Survival was illustrated by Kaplan-Meier curves; survival differences between groups were examined with log-rank test. All data were analyzed for significance using GraphPadPrism 6 software (San Diego, CA, USA); a probability level < 0.05 was considered significant throughout the analysis.

## SUPPLEMENTARY MATERIAL FIGURES AND TABLE


