# Supplementary material for: miR-340 predicts glioblastoma survival and modulates key cancer hallmarks through down-regulation of NRAS
Source: Oncotarget. 2016 Jan 21;7(15):19531–47. doi: 10.18632/oncotarget.6968 (PMC4991399; doi:10.18632/oncotarget.6968)
Supplement: Supplementary file 1 [file oncotarget-07-19531-s001.pdf]

miR-340 predicts glioblastoma survival and modulates key cancer hallmarks through down-regulation of *NRAS*

Supplementary Materials

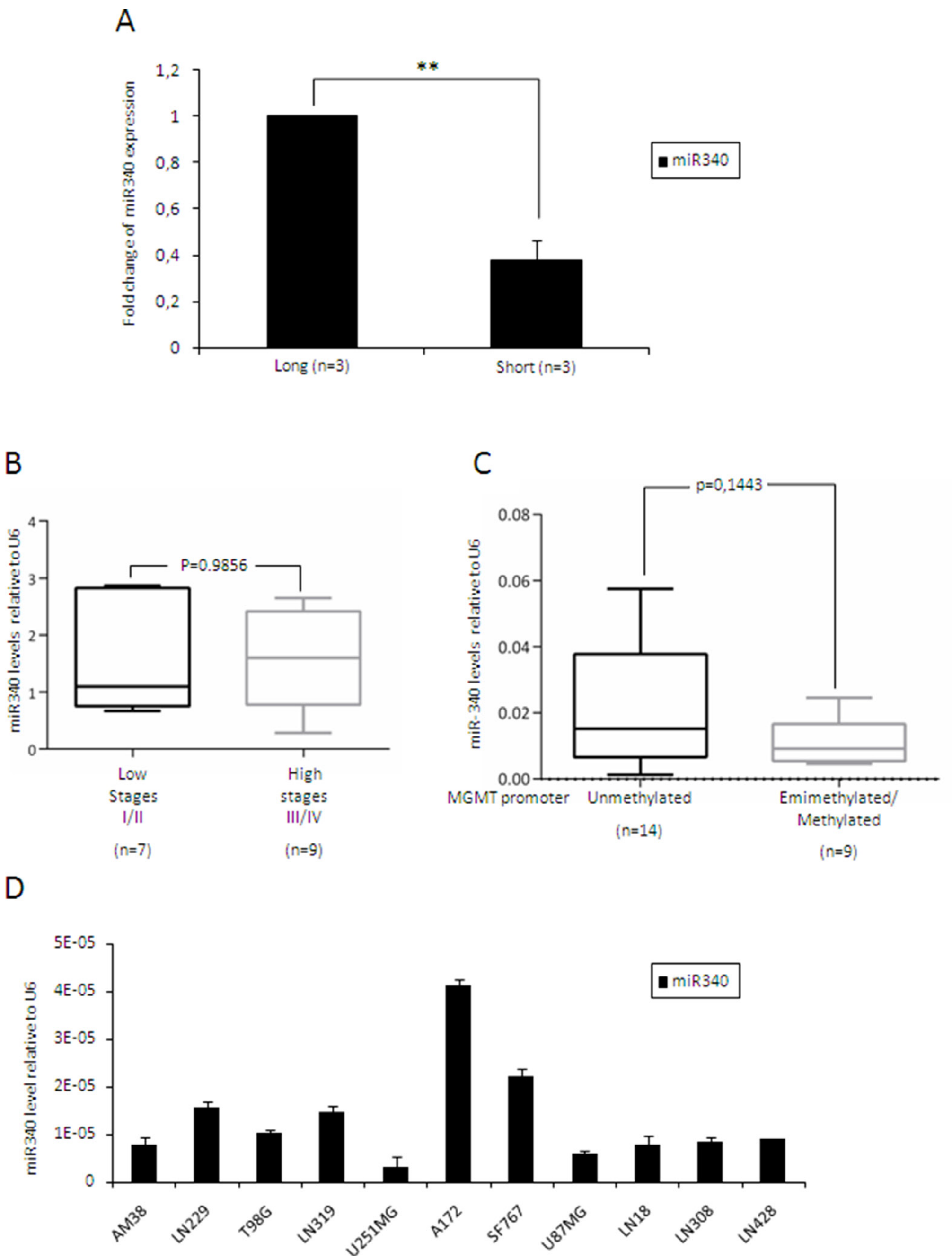

Supplementary Figure S1: miR-340 expression in glioblastoma. (**A**) Array validation. Real-time PCR of miR-340 from 3 LTS and 3 STS.miR-340 was down-reregulated in LTS vs STS. (**B**) miR-340 expression was evaluated in FFPE tissue from 7 Low Stages, and 9 High Stages glioma patients. The correlation between miR-340 expression and glioma stages did not result statistically significant. (**C**) miR-340 expression was evaluated in FFPE tissue from 14 Unmethylated, and 9 Emimethylated/Methylated glioma patients in MGMT promoter. The correlation between miR-340 expression and methylation status of MGMT promoter did not result statistically significant. In (A) (B) (C) *P* was calculated using Student's *t*-test. ***p* < 0.01.Transcript level was normalized against U6. (**D**) Endogenous miR-340 levels analyzed by real-time PCR in 11 different glioblastoma cell lines. Transcript levels was normalized against U6. The data are representative of three independent experiments. Data are mean values ± SD from three independent experiments.

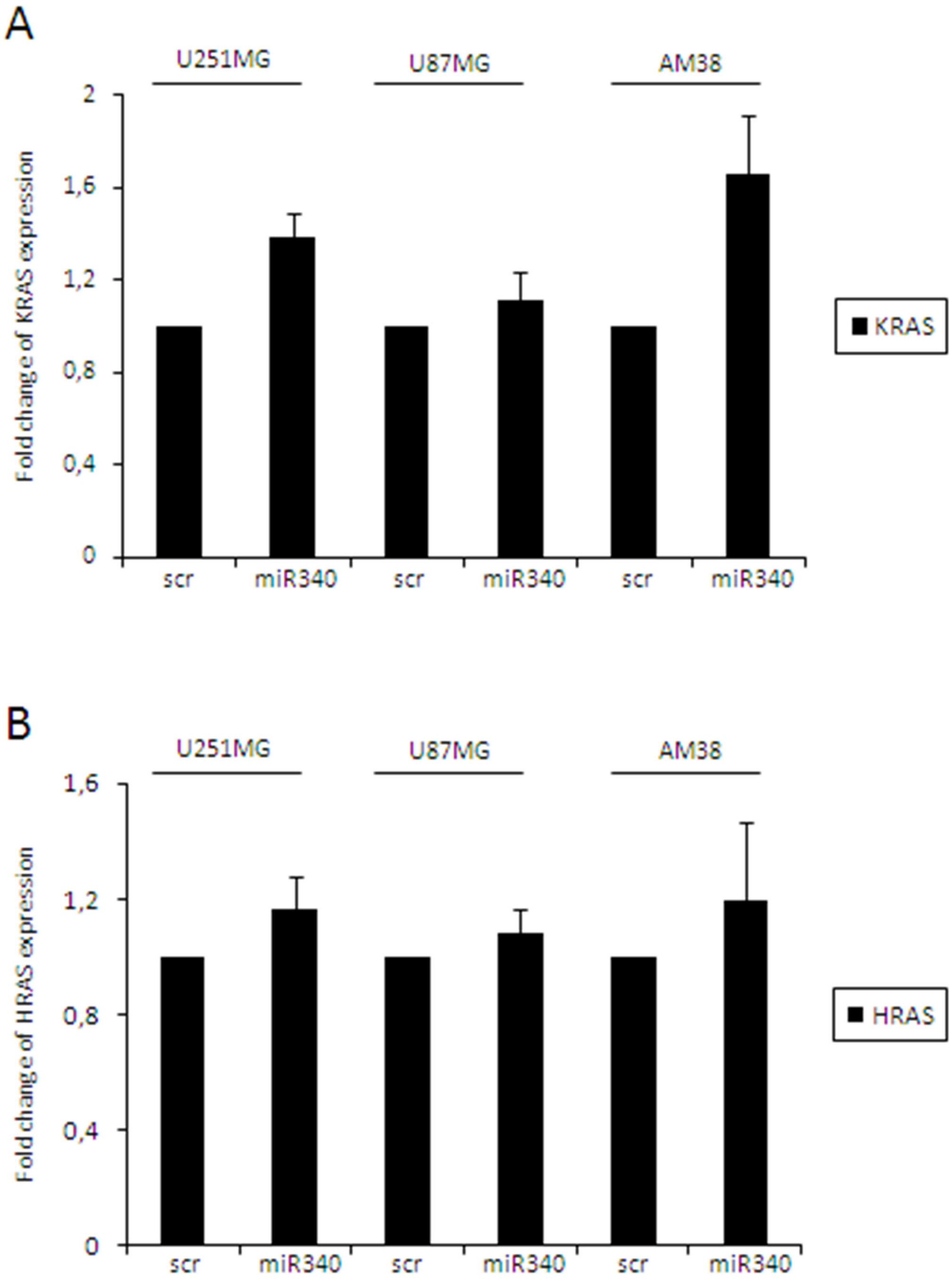

Supplementary Figure S2: miR-340 selectively targets *NRAS* mRNA. Glioblastoma cell lines (U251MG, U87MG and AM38) were transfected with a scrambled miRNA sequence or with miR-340 for 72 h. Real-time PCR was performed to analyze *KRAS* and *HRAS* mRNA levels. Transcripts levels were normalized against actin.The data are representative of three independent experiments. Data are mean values ± SD from three independent experiments.

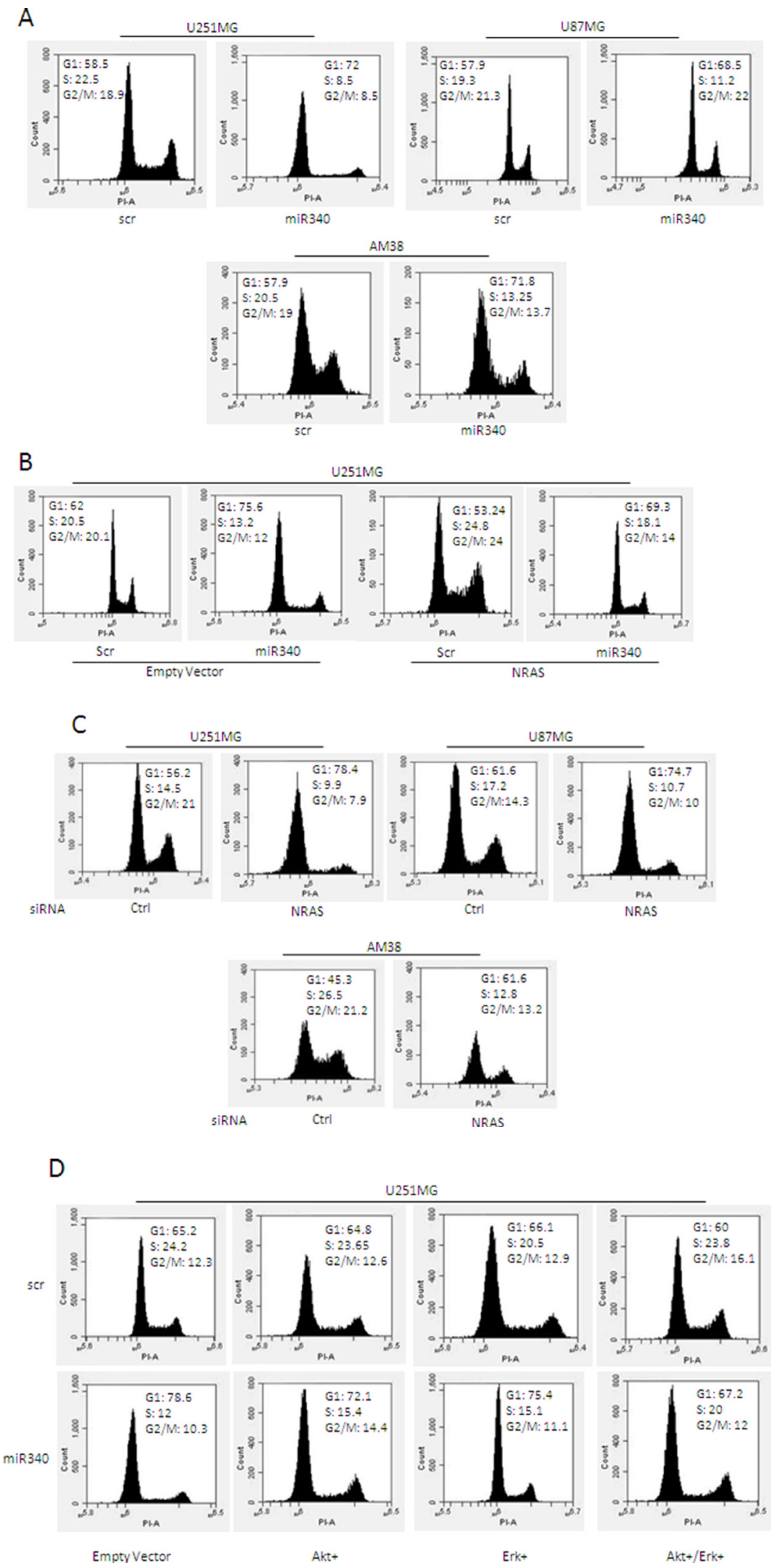

Supplementary Figure S3: Qualitative analysis of cell cycle.Plots for qualitative representation of cell cycleanalyzedby flow cytometry after propidium iodide staining are provided for: (**A**) glioblastoma cell lines (U251MG, U87MG and AM38) transfected with miR-340 or with a control scrambled miRNA sequence; (**B**) U251MG cells co-transfected with miR-340 and either a vector carrying NRAS lacking 3′UTR or a control; (**C**) U251MG, U87MG, and AM38 cells transfected with a control siRNA or with a specific siRNA targeting NRAS; (**D**) U251MG cells co-transfected with miR-340 and dominant positive mutants constructs of ERK and AKT, alone or in combination, or with control vector.

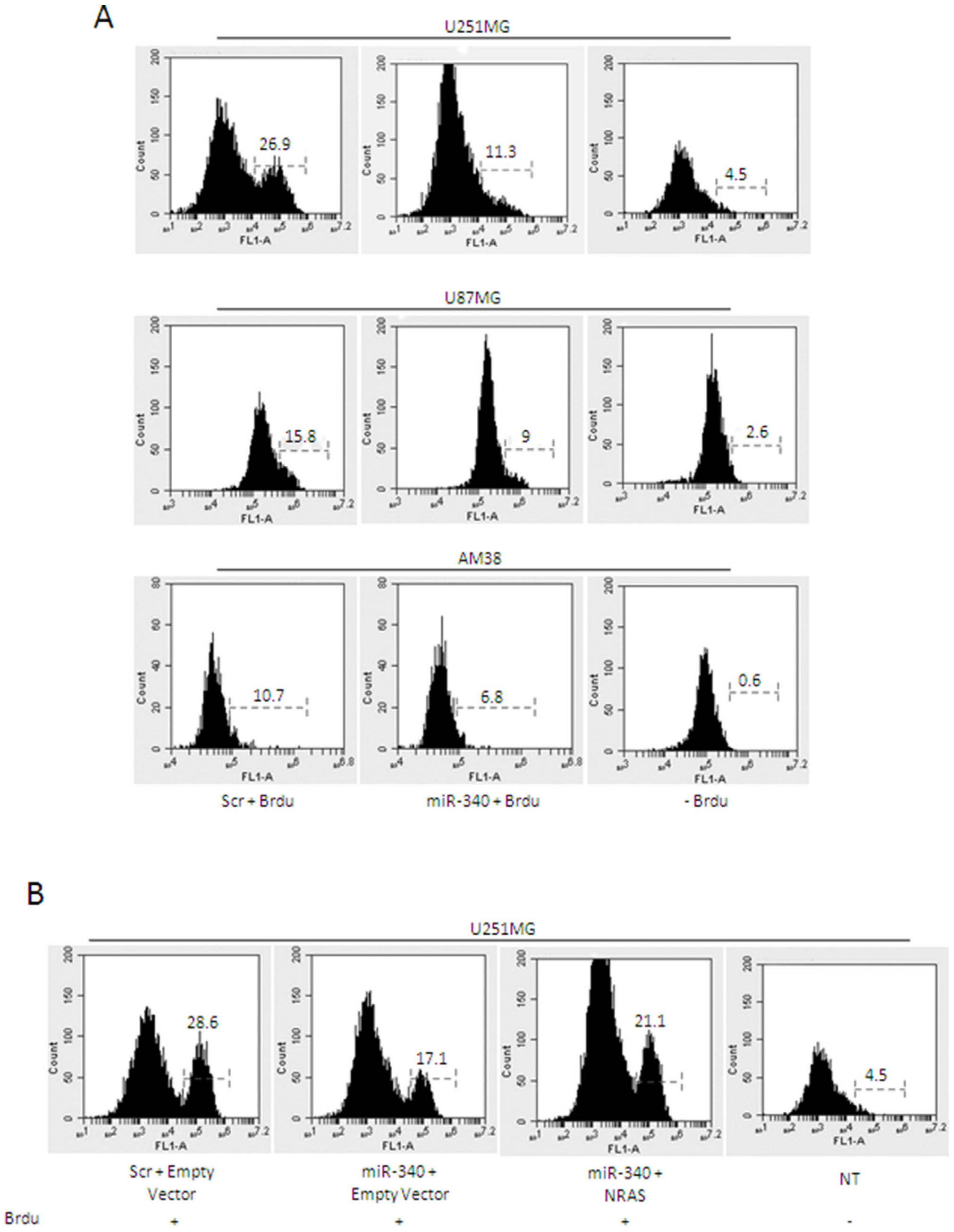

Supplementary Figure S4: Qualitative analysis of BrdU incorporation assay. Plots for qualitative representation of BrdU incorporation assayanalyzedby flow cytometry after BrdU incorporation and anti BrdU-Fluorescein antibody staining are provided for: (**A**) glioblastoma cell lines (U251MG, U87MG and AM38) transfected with miR-340 or with a control scrambled miRNA sequence; (**B**) U251MG cells co-transfected with miR-340 and either a vector carrying NRAS lacking 3′UTR or a control.

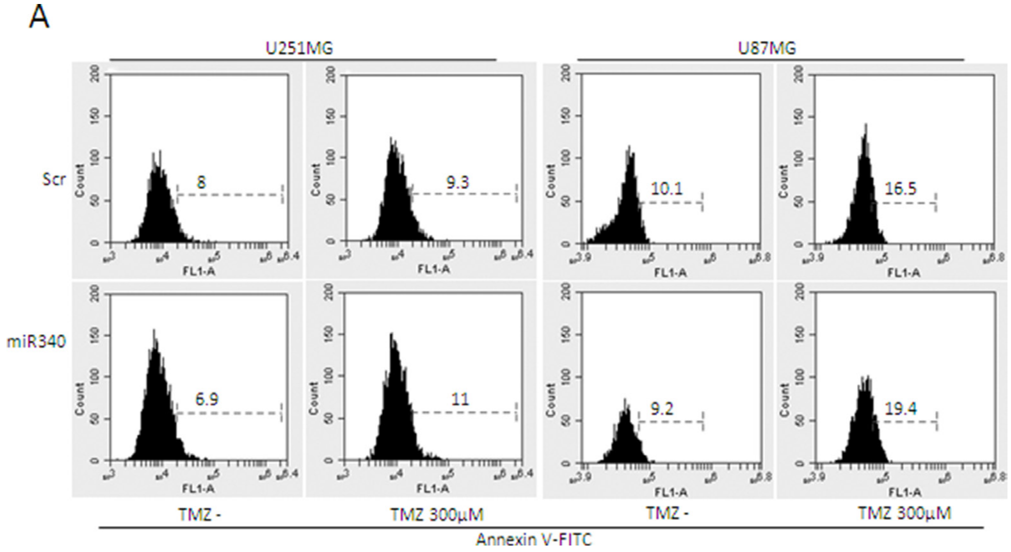

Supplementary Figure S5: Qualitative analysis of Annexin V assay. Plots for qualitative representation of Annexin V assayanalyzedby flow cytometry after Annexin V-FITC staining are provided for: (**A**) glioblastoma cell lines (U251MG, and U87MG) transfected with a scrambled miRNA sequence or miR-340 for 24 h, and then treated with 300 µM TMZ for 24 h.

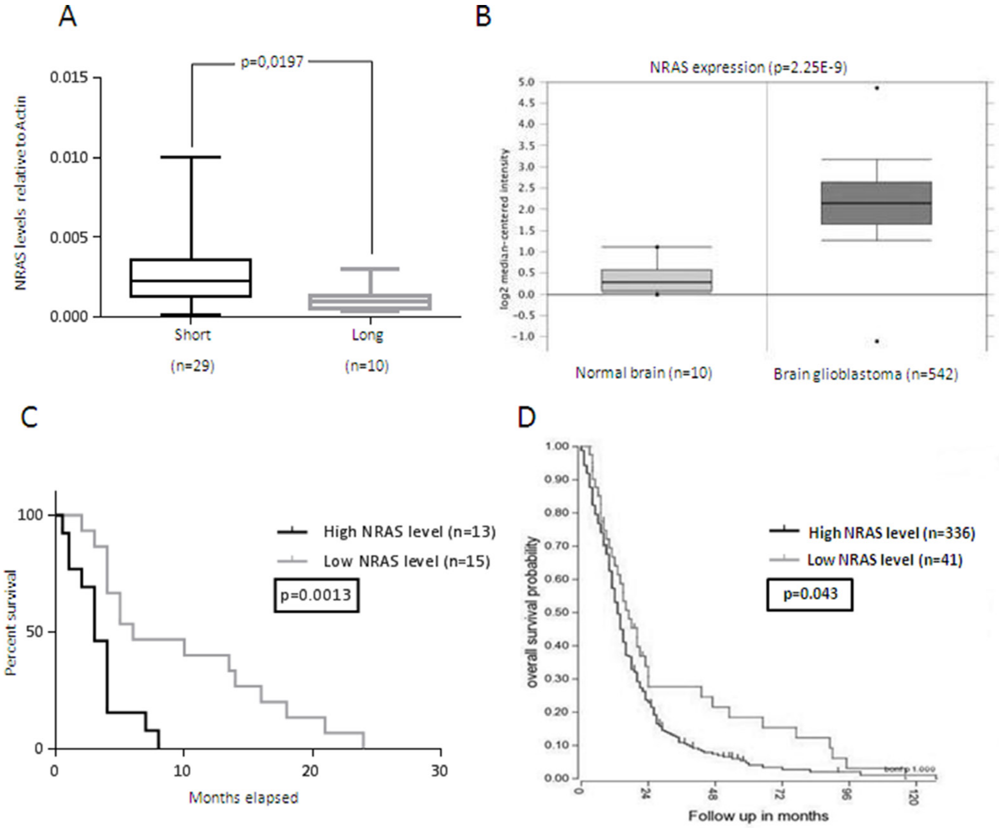

Supplementary Figure S6: ***NRAS*** **is down-regulated in GBM and correlates with prognosis of GBM patients.** Analysis was performed on three independent patient cohorts from our hospital (**A** and **C**), from the ONCOMINE database (**B**) and from the R2.aml database (**D**). *NRA*S expression in: (A) FFPE tissues from 10 LTS and 29 STS glioblastoma patients; (B) 10 normal brain and 542 glioblastoma specimens from ONCOMINE. A significant increase in *NRAS* expression was identified in STS vs LTS (A) and in glioblastoma vs normal brain (B). *NRAS* expression level was assessed by real-time PCR. Transcript level was normalized against actin. An arbitrary cut-off of 12 months was used to stratify patients. *P* was calculated using Student's *t*-test. $P < 0.05$ was considered significant. (C, D) Kaplan-Meier survival curve analysis of the correlation between *NRAS* and overall survival of 13 highly and 15 poorly *NRAS*-expressing glioblastoma patients from our hospital (C) and of 336 highly and 41 poorly *NRAS*-expressing glioblastoma patients from the R2.aml database (D). Low *NRAS* expression predicted better prognosis in GBM patients. The patients were assigned to the high- or low-*NRAS* expression group according to R2.aml database. *P* was calculated using Log-Rank test. $P < 0.05$ was considered significant.

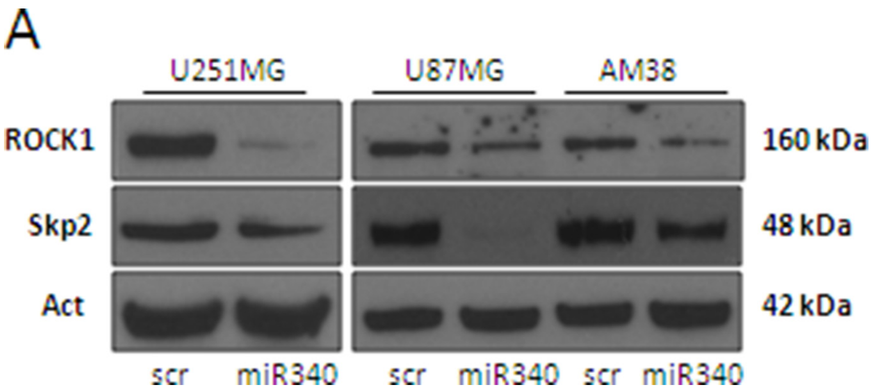

Supplementary Figure S7: miR-340 decreases SKP2 and ROCK1 in glioblastoma. Glioblastoma cell lines (U251MG, U87MG and AM38) were transfected with a scrambled miRNA sequence or miR-340. Western blotting was performed to analyze SKP2 and ROCK1 protein levels. Western blot analyses are from representative experiments. Actin was used as the loading control. The experiments were repeated at least three times.

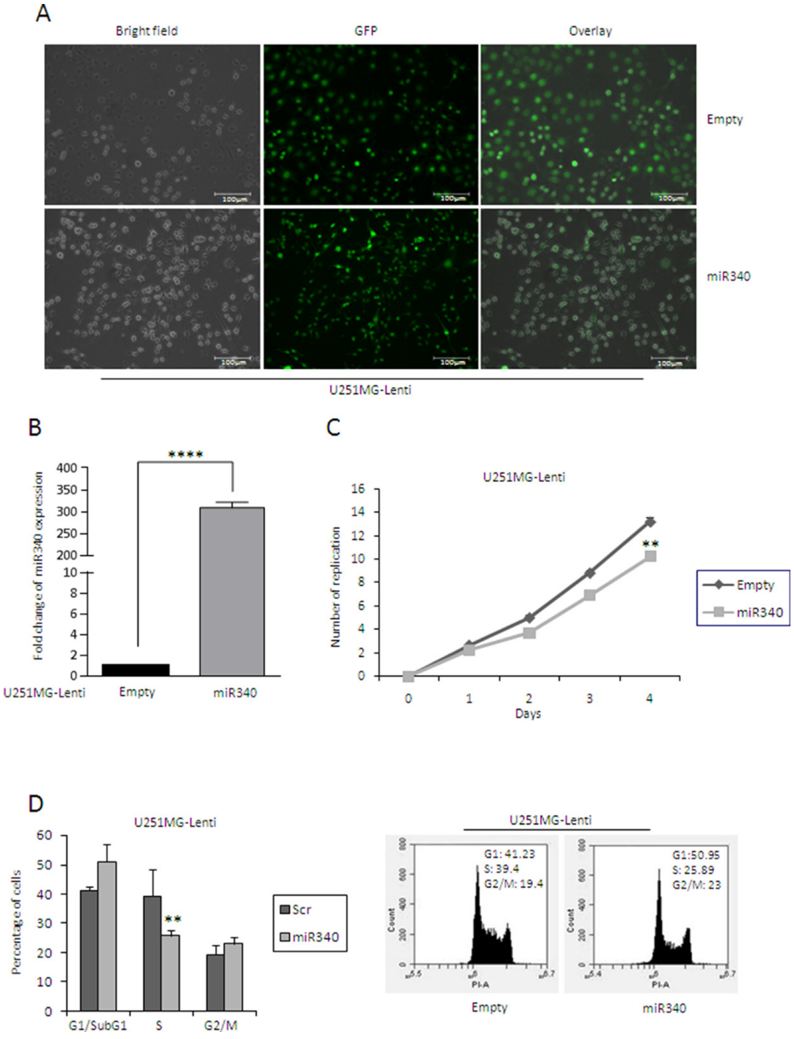

Supplementary Figure S8: U251MG lentiviral vector transduction and selection. (**A**) U251MG cells were stably transduced with a lentiviral vector encoding mature miR-340 or a control sequence, together with GFP and a puromycin-resistance gene (MOI = 20). Stably-transduced clones were isolated by GFP expression in a medium supplemented with puromycin (original magnification 10x; scale bar 100 µm). Expression levels of miR-340 were checked by real-time PCR. The transcript level was normalized against U6 (**B**). Cell proliferation and cell cycle were analyzed respectively by MTT assay (**C**) and PI staining and flow cytometry (**D**). miR-340stably expression blocked cell cycle and decreased cell proliferation.Presented data are mean values ± SD from three independent experiments. *P* was calculated using Student's *t*-test. $^{**}p < 0.01$;$^{****}p < 0.0001$.

Supplementary Table S1: miRNAs differentially expressed between long-(LTS) and short-(STS) term glioblastoma patients

	Unique id	Ratio of geom means Long vs Short survivors
**1**	hsa-mir-193b	0.491352201
**2**	**hsa-mir-340**	**1.505219391**
**3**	hsa-mir-19b	1.537663509
**4**	hsa-mir-20a - b	1.880932671
**5**	hsa-mir-219-5p	1.905150526
**6**	hsa-mir-137	2.235717499
**7**	hsa-mir-129-3p	2.644557823

Fold change values were generated from the median expression of the miRNAs in the groups compared. Statistical comparisons were done with the GENESPRING ANOVA tool, predictive analysis of microarray and the significance analysis of microarray software.
